# SOM0226, a repositioned compound for the treatment of TTR amyloidosis

**DOI:** 10.1186/1750-1172-10-S1-P9

**Published:** 2015-11-02

**Authors:** Núria Reig, Salvador Ventura, Maria Salvadó, Josep Gámez, Raúl Insa

**Affiliations:** 1 SOM Biotech, R&D, 08028, Barcelona, Spain; 2 Universitat Autònoma de Barcelona, Institute of Biotechnology and Biomedicine, 08193, Bellaterra, Spain; 3 Hospital Universitari Vall d'Hebron, Neurology, 08035, Barcelona, Spain

## Background

Transthyretin amyloidosis is a protein aggregation disorder caused by deposition of transthyretin, a tetrameric plasma protein in charge of the transport of thyroxin and retinol in plasma and cerebrospinal fluid. Kinetic stabilization of the TTR tetramer has proved to be a valid therapeutic strategy to prevent the release of unstable TTR monomers and their aggregation into oligomers and amyloid fibrils. SOM0226 is tolcapone, a repositioned compound with a newly identified activity as a potent TTR stabilizer and TTR fibril disruptor.

## Methods

A proof of concept Phase IIa clinical trial has been conducted in diagnosed Familial Amyloid Polyneuropathy (FAP) patients, asymptomatic carriers and healthy volunteers with the objective to determine whether treatment with SOM0226 stabilizes plasmatic TTR. The trial has been conducted at the Hospital Universitari Vall d'Hebron (Barcelona) and has involved 6 healthy individuals and 15 asymptomatic carriers with mutations in the TTR gene and FAP patients at different stages of disease progression. It was an interventional open label study organized in two phases separated by a washout period of 6 weeks. During the first phase, patients were administered a single dose of SOM0226 (200mg) and blood was collected at different times for determination of drug levels and TTR stabilization activity, which was the primary efficacy endpoint of the study. The second phase was similar but involved multiple doses of 100mg of SOM0226: one every 4 hours up to a total of 3 doses. TTR stabilization was measured in plasma samples using an immunoturbidity method after urea denaturation and crosslinking with glutaraldehyde. Safety endpoints were also determined at baseline and at the end of each phase.

## Results

Treatment with a single oral dose of 200mg or three doses of 100mg SOM0226 induce a clear and robust stabilization of plasmatic TTR in all patients studied, allowing the protection of 100% of TTR present in the plasma samples. This activity is maximal after 2 hours of dosing and clearly correlates with drug levels in plasma. The studied administration regime has demonstrated to be safe, with no adverse events related to the investigational product observed. Moreover, significant clinical or analytical hepatotoxicity has not appeared in any patient.

## Conclusion

SOM0226 is capable of stabilizing all TTR present in plasma samples in all patients studied, supporting further development of SOM0226 for the treatment of TTR amyloidosis. As a repositioned drug, SOM0226 (tolcapone) can bypass much of the early cost and time needed to bring a drug to market and bears the potential to become the most potent TTR stabilizer in the market to prevent the progression of the different forms of TTR amyloidosis.

